# Co-designing Healthy Living after Cancer Online: an online nutrition, physical activity, and psychosocial intervention for post-treatment cancer survivors

**DOI:** 10.1007/s11764-022-01284-y

**Published:** 2022-11-14

**Authors:** Morgan Leske, Bogda Koczwara, Jason Blunt, Julia Morris, Elizabeth Eakin, Camille E. Short, Anthony Daly, Jon Degner, Lisa Beatty

**Affiliations:** 1 College of Education, Psychology, and Social Work, Flinders University, Adelaide, SA Australia; 2 College of Medicine and Public Health, Flinders University, Adelaide, SA Australia; 3 Department of Medical Oncology, Southern Adelaide Local Health Network, Adelaide, SA Australia; 4 Cancer Council SA, Eastwood, SA Australia; 5 School of Public Health, Faculty of Medicine, University of Queensland, Brisbane, QLD Australia; 6 Melbourne Centre for Behaviour Change, Faculty of Medicine, Dentistry and Health Sciences, The University of Melbourne, Melbourne, VIC Australia; 7 Cancer Voices South Australia, Kensington Park, SA Australia

**Keywords:** Cancer survivors, Lifestyle intervention, Co-design, Digital intervention

## Abstract

**Purpose:**

The aim of the present study was to co-design Healthy Living after Cancer *Online* (HLaC *Online*), an online intervention supporting cancer survivors to set and meet their healthy living goals.

**Methods:**

Adapted from an initial telephone-delivered Healthy Living after Cancer program, wireframes (PDF black and white mock-ups) of the proposed online program were presented in a series of focus groups and interviews to our stakeholder group, which consisted of cancer survivors, oncology healthcare professionals, and representatives from cancer support organisations. Stakeholders were prompted for feedback on the wireframe and given end-user scenarios to encourage deeper engagement with the co-design process. Transcriptions underwent thematic analysis to determine which features of the program needed change or expansion.

**Results:**

27 participants took part in one of 8 focus groups or 10 interviews. Five themes were identified relating to (a) website design elements, (b) promoting and maintaining long-term engagement, (c) relatability and relevance, (d) navigating professional support, and (e) family and peer support. Recommended changes, such as simple activities and guidance videos, were integrated into the HLaC *Online* prototype.

**Conclusions:**

Involving end-users in the co-design process ensured the intervention’s relevance and specificity to the needs of cancer survivors. Next steps include feasibility testing the prototype, prior to commencing a national randomised control trial of HLaC *Online*.

**Implications for Cancer Survivors:**

HLaC *Online* aims to support cancer survivors to improve their quality of life by making healthy lifestyle changes in their physical activity, healthy eating, weight management, mental health, and fatigue management.

**Supplementary Information:**

The online version contains supplementary material available at 10.1007/s11764-022-01284-y.

Engaging in a healthy lifestyle after cancer, including regular physical activity and adequate nutrition, can reduce the risk of mortality, cancer recurrence [1–3], and comorbidities [4]. Further, healthy lifestyle behaviours have been shown to mitigate some of the challenging impacts of cancer and its associated treatments, including improving cancer-related fatigue [5] and reducing psychological distress [6]. Despite these benefits, many Australian cancer survivors are not meeting the healthy lifestyle recommendations outlined by national cancer support organisations [7, 8]. A report from Tollosa et al. [9] using data from the Australian Longitudinal Study on Women’s Health showed that 41%, 36%, and 85.1% of female cancer survivors were not engaging in the health recommendations for physical activity, fruit intake, and vegetable intake, respectively. More recently, Elder-Robinson et al. [10] investigated health behaviours of Australian cancer survivors in rural and remote areas, demonstrating that up to 27% had reduced their fruit and vegetable intake and 70% had reduced their physical activity since their cancer diagnosis.

Face-to-face interventions have demonstrated efficacy in improving health behaviours; however, these interventions are not routinely implemented in clinical care at the completion of cancer treatment [11]. This evidence-practice gap has emerged due to implementation barriers experienced at the three levels of cancer survivorship care: (1) organisational level barriers, such as the cost and lack of reimbursement for delivering interventions, no established pathways for managing referrals and follow-ups, and absence of specialised staff to deliver the intervention; (2) provider level barriers, including limited time, competing priorities, not aware of existing programs, and not self-identifying as the right person to provide advice; and (3) consumer-level barriers, such as lack of guidance and support, not understanding the benefits of participating in health programs, low engagement in interventions due to competing priorities, and/or high levels of fatigue [12–15]. Cancer survivors who live in rural and remote areas of Australia experience additional accessibility barriers, imposed by the time and financial costs of travel [16]. Finally, the ongoing social distancing restrictions associated with the COVID-19 pandemic have reduced practitioners’ ability to address health concerns and behaviours in face-to-face appointments [17]. These barriers highlight the importance of utilising cost-effective and accessible delivery modalities to increase the reach and availability of health interventions.

The telephone has previously be investigated as an accessible and acceptable modality for health interventions [18]. One such Australian intervention was the 6-month telephone-delivered program Healthy Living after Cancer [19]. The intervention targeted goal setting, physical activity, nutrition, weight loss, and behavioural maintenance strategies. Healthy Living after Cancer was delivered in several states by Cancer Council, an Australian not-for-profit cancer support organisation, using their existing telephone support infrastructure. While the program yielded significant clinical benefits to participants, including improvements in physical activity, dietary behaviours, physical quality of life, and cancer-related symptoms, sustainability barriers were encountered [19].The intervention was resource intensive, and Cancer Councils were unable to continue providing the program after the trial ceased. Furthermore, feedback from participants suggested that while many were satisfied with the telephone delivery, it did not suit all users’ preferences. Some participants experienced challenges specific to the telephone delivery, including difficulties scheduling calls, feeling rushed, and a decrease in motivation when calls shifted from weekly to monthly delivery as per intervention protocol [20]. Therefore, other delivery modalities needed to be explored to improve sustainability of the program.

Digital health modalities, including patient portals, online support tools, and mobile applications, have emerged as a cost-effective and accessible way to deliver health-related services [21, 22]. Digital health modalities enable participants to self-tailor their information access and can integrate dynamic elements to support users to establish and achieve their health-related goals [23]. Adapting the Healthy Living after Cancer intervention into a digital health modality therefore has the potential to enhance the program’s reach, flexibility, scalability, and long-term sustainability.

While approximately twenty English digital health interventions have been developed to address health behaviours in cancer survivors in the last decade [16, 24–27], none have previously utilised a co-design process. Co-design involves end-users at each stage of intervention development, resulting in an intervention that is both sensitive to consumer’s specific needs and preferences and follows best-practice principles for consumer-led development of interventions [28, 29]. The Healthy Living after Cancer *Online* (HLaC *Online*) research team commenced the co-design process with a group of stakeholders to adapt the program iteratively from its telephone-delivered format using a five-phase Design Thinking Research Process, comprised of empathising, defining, ideating, prototyping, and testing [30]. The first round of stakeholder engagement addressed the first two phases (emphasising and defining). This round of stakeholder engagement [31] found that the HLaC *Online* program should target not only physical activity, healthy eating, and weight management, but also offer support for mental health, fatigue management, and peer support. Additionally, stakeholders reported that the intervention should offer a flexible format and long-term accessibility.

The present study aimed to conduct the third and fourth phase of the co-design process—ideate and prototype—through a second round of stakeholder engagement. This round involved presenting and receiving feedback on a wireframe, that is, a visual guide representing a skeletal framework containing all the proposed content of HLaC *Online*. Wireframes are an established methodology for ideating and prototyping interventions and have been used in the co-design of digital health interventions for people with cancer [32], knee osteoarthritis [33], and heart failure [30]. Specifically, the second round of stakeholder engagement sought to clarify cancer survivor’s needs for healthy living guidance and support, whether these needs would be met by the new program, identify potential barriers for program engagement, and develop strategies to best support users.

## Methods

### Participants

Participants were recruited through two sources. First, stakeholders from the first round of engagement [31] were invited to return for the second round of stakeholder engagement. These participants included Australian cancer survivors, oncology healthcare professionals, and non-government organisation cancer support representatives. Second, additional participants were identified and invited through snowball sampling of the stakeholder participants’ networks. Reasons for not returning for the second round of stakeholder engagement for cancer survivors included no longer being interested (*n* = 4), engagement not occurring at a good time (*n* = 1), or personal reasons (*n* = 1). Three cancer survivors did not respond to contact. Reasons for not returning for healthcare professionals and cancer support representatives included no longer being interested (*n* = 2), no longer working in cancer (*n* = 1), or cancelling after focus group was rescheduled (*n* = 1).

### Wireframe

The wireframe of HLaC *Online* was developed based on the telephone-delivered Healthy Living after Cancer program [19] and the findings from the first round of co-design [31] (see Online Resource 1). The wireframe comprised nine modules, including five from the original telephone-delivered program (goal setting, physical activity, healthy eating, maintaining a healthy weight, staying on track) and four newly developed modules (mental health, fatigue management, finding the new normal, and peer support). Each module consisted of psychoeducation, activities based on the Social Cognitive Theory [34] constructs of self-efficacy, outcomes expectancies, and social support (e.g., goal setting, self-monitoring, problem solving, self-reward, and social support), and links to reputable resources (e.g., non-governmental cancer support organisations websites, such as Cancer Council Australia). The mental health module included activities based on cognitive behavioural therapy (e.g., thought records, and identifying and challenging unhelpful thoughts) and mindfulness relaxation. Finally, the finding of the new normal and the peer support modules included survivor testimonial videos.

### Data collection

All stakeholders completed informed consent before participating. Focus groups (*M* = 87 min, *SD* = 24) and interviews (*M* = 72 min, *SD* = 10) were conducted between October and December 2020. Due to ongoing social distancing requirements of COVID-19 restrictions, stakeholders participated either via small face-to-face focus groups (*n* = 2–3 per group), an online focus group, or interview held on a secure videoconferencing platform, Webex. Two cancer survivor stakeholders were interviewed via telephone due to internet difficulties. Stakeholders were provided with a summary of key findings from the first round of stakeholder engagement and presented with the HLaC *Online* wireframe.

Stakeholders were invited to provide feedback on the new content, along with one of the original modules from the telephone-delivered Healthy Living after Cancer, which was randomly selected for each focus group and interview. A semi-structured topic guide was utilised to facilitate feedback (see Online Resource 2), along with a persona task to facilitate discussion about how potential users might use the program and how they could best be supported. This task involved the stakeholders developing a hypothetical user of the program and included a description of their name, age, gender, cancer diagnosis, and healthy living goals (see Online Resource 3 for an example).

### Data analysis

Audio recordings from the focus groups and interviews were transcribed verbatim. Transcriptions underwent inductive thematic analysis using the qualitative data analysis software, NVivo 12. Inductive thematic analysis was chosen to determine which features of the program should be considered for change or expansion based on the stakeholder’s feedback. Two authors (ML, JB) independently undertook thematic analysis on a subset of the transcripts (*n* = 8) to develop a preliminary coding framework. The coding framework was refined through discussion with authors with extensive qualitative research experience (BK and LB) to finalise and diagram the themes and subthemes. The final coding framework was then used to analyse all transcripts by a single author (ML).

## Results

### Participants

A total of 29 stakeholders (14 cancer survivors, 13 healthcare professionals, and 2 cancer support representatives) participated in one of seven focus groups or nine interviews, resulting in 16 transcripts. This equated to 71% of our original stakeholder group continuing their involvement from Round 1, along with one additional healthcare professional and one cancer support representative.

The majority of cancer survivors were female (*n* = 8, 57.1%) and aged between 44 and 81 years (*M* = 61, *SD* = 12.17). The most common cancer diagnosis was breast cancer (*n* = 6, 42.9%), followed by prostate cancer (*n* = 3, 21.4%), rectal cancer (*n* = 2, 14.3%), cervical cancer (*n* = 1, 7.1%), and Hodgkin’s lymphoma (*n* = 1, 7.1%).

Most healthcare professionals were nurses (*n* = 7, 53.8%) but included medical oncologists (*n* = 2, 15.4%), a clinical psychologist (*n* = 1, 7.7%), and a physiotherapist (*n* = 1, 7.7%). Cancer support representatives included a support group representative and a representative from Cancer Council SA’s support services.

### Overview of themes and subthemes

**Fig. 1:**
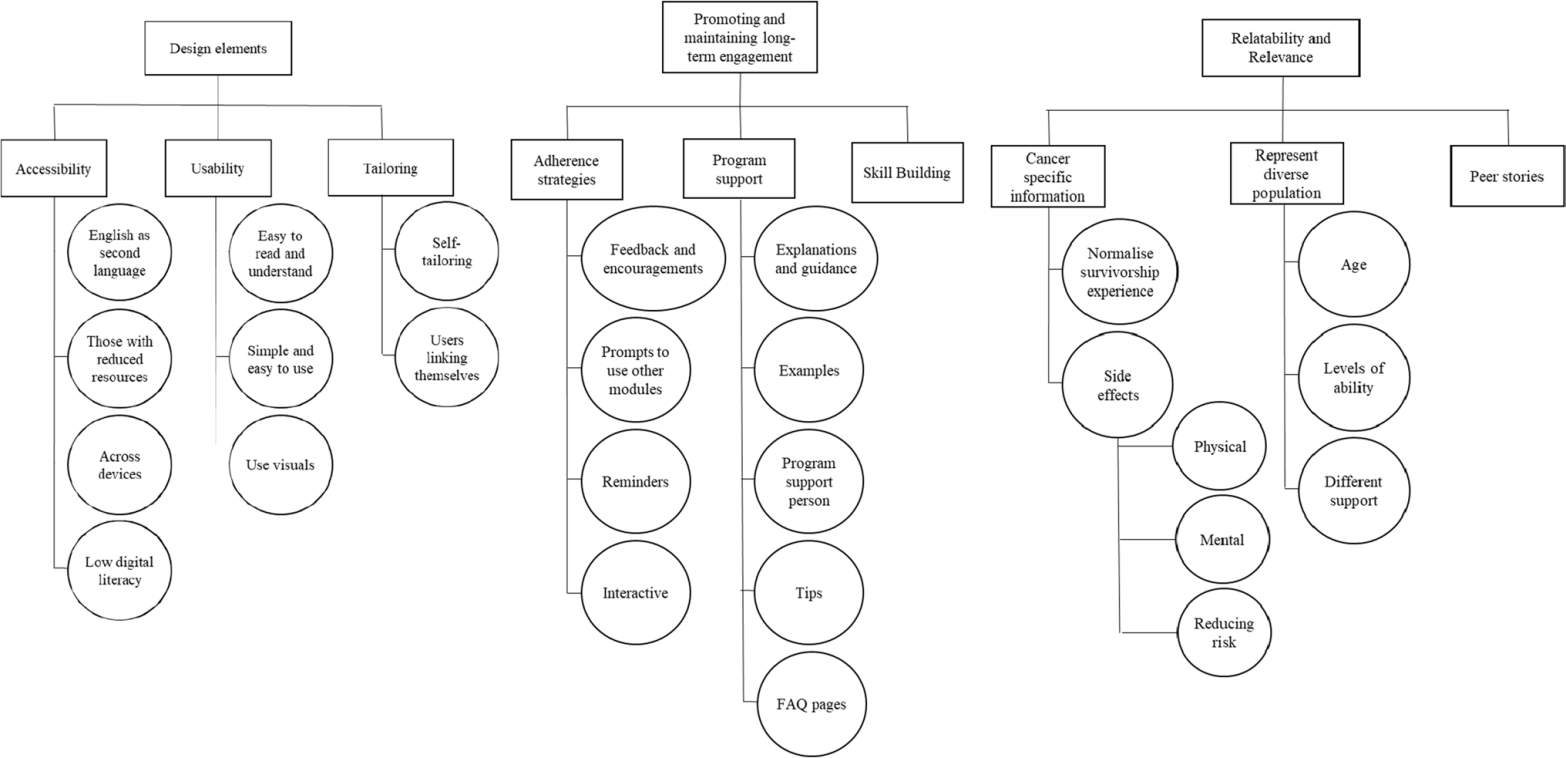
Stakeholder themes from second round of co-design

A total of 5 themes and 16 subthemes emerged from the thematic analysis. Overall, the wireframe received positive feedback from participants. All participants agreed that the program addressed key concerns of cancer survivors and praised the addition of modules based on their previous feedback. Five themes emerged relating to (a) website design elements, (b) promoting and maintaining long term engagement, (c) relatability and relevance, (d) navigating professional support, and (e) family and peer support (see Fig. 1).

### Theme 1: website design elements

As Fig. 1 shows, this theme related to how the web-program will be designed to increase accessibility, usability, and the ability to self-tailor the program.

#### Accessibility

One key subtheme to emerge was that HLaC *Online* must be developed in a way that ensures it is *accessible* to the diverse cancer survivor population. All stakeholder groups strongly endorsed that the program should be designed in a way to accommodate different devices and levels of digital literacy. Cancer survivors more frequently endorsed the use of different language settings so that the program is accessible to those for whom English is their second language.

*“I come from basically Pakistan, and I speak another language. So, it would be good, when you’re living here if you can find somebody who can speak your language also. If you can’t speak English, which is, you know, if you’re just alone by yourself and it’s all English and you do not have the information… that would be a good idea to put in other languages, or to show that everybody’s included” *(CS03).

In comparison, the healthcare professionals frequently highlighted that any suggested healthy lifestyle changes, such as the type of exercise, must be accessible to users with limited resources. This was especially important when considering potential users who live in rural and remote communities.

*“With the aerobic work, a lot of people only really have walking as their accessible option because they can’t get to a pool, they’re not into jogging, and they can’t ride a bike. So, I think you need to sort of perhaps, particularly focus on the walking side of aerobic because that is the again that was easily accessible for the majority of people”* (HCP06).

#### Usability

It was important to stakeholders that HLaC *Online* is *user friendly*; the website must be simple to use and easy to navigate, and information provided both easy to read and understand. Stakeholders promoted the use of visuals, such as videos, images, and diagrams, to reduce the reading burden on users.

*“The most important resource would be actual patient experience, you know. Short videos is what I would sort of you know would recommended given this, the nature of the situation as well as how technology is taken over. To reading through lots, through lots and lots of text, I don’t think they have much of an uptake overall”* (HCP05).

The wireframe received mixed feedback as to whether these needs were met. Overall, the stakeholders thought the program appeared easy to use; however, some activities may have been too complex for a self-guided program. One common piece of feedback from all groups was the need to simplify the thought record, where users can record and challenge their thoughts.

*“I just wonder if it’s too complicated. I think the mindfulness, I think is something that people can engage in quite easily. And this to me, like I get it, but I’m wondering how many people will engage in it or it’ll just be a bit too complicated”* (HCP02).

#### Self-tailor

It was important to all stakeholder groups that HLaC *Online* offer users the ability to self-tailor the information, such that they can choose when and how they access the information and complete activities.

*“It’s fine because I think if think people will just read it, look at it and read it and choose the one that pertains to them at that time. And for some people, fatigue management might be first and for someone else it might be exercise. So, just have them all and then people will do what they want to do anyway”* (HCP11).

Cancer survivors more frequently suggested that the program be designed in a way that users could print and complete activities by hand. This was only mentioned once in the healthcare professional group and was not mentioned by cancer support representatives.

*“Those might be something that we can look at where they can download the page for instance because some people are writers too. Some people are, not a lot of us are keyboard warriors and a lot of people enjoy writing on something instead of a keyboard”* (CS11).

### Theme 2: promoting and maintaining long-term adherence

This theme related to feedback about how to engage users and maintain long-term adherence to the program and health behaviour changes.

All groups frequently endorsed the use of strategies to increase the adherence and usage of the program. During the persona task, a common description of a potential user was someone who is initially very engaged with the program and making healthy changes; however, this behaviour would gradually taper off. For example:

*“He initially he would be in it for a number of weeks and then he has to be obviously encouraged to continue it. And that’s probably where he might get off track. But, you know, in the initial stage, you’ll probably be all gung ho about it. But in the weeks down the track he might get a bit blasé, or anything are not happening quick enough at all certainly falls into a trap. Getting into the junk food again”* (CS01).

Common recommendations to address these issues and increase engagement included using adherence strategies, promoting skill building, and providing program support. Each of these is outlined in detail below.

#### Adherence strategies

A variety of strategies to increase engagement and adherence with the program were suggested, including feeding back previously input information into later activities, encouragements throughout the program, prompts to use other areas of the program, interactive elements (e.g., activities, videos, audio files, and animations), and reminders to use the program. For example:

*“Do they get the results of their trackers? Would that be included in the email? So, you’ve done so many steps. You know, we encourage great work. We encourage you to do and more or loss this much weight. So, it’s like data being fed back to them as well as encouragement to keep going”* (HCP04).

There was mixed feedback for the frequency of reminders to use HLaC *Online*. However, the majority of stakeholders agreed that participants should be engaging with the program at least once a week, and reminders should be sent accordingly. One cancer survivor and one healthcare professional suggested this could be tailored, with the user able to determine the frequency of reminders.

#### Program support

All stakeholder groups suggested some level of guidance on *how* to use the program, although this was more frequently endorsed by cancer survivors. Cancer survivors’ most frequently suggested form of guidance involved having a person to discuss the content with, either via regular phone calls or someone to contact when they require assistance.

*“You could have regular phone calls from a cancer council nurse. Or text messaging service that help him. See how he's doing with his goals and helping sort of just keep him a bit motivated”* (CS07).

*“I think it’s pretty comprehensive and easy to use, but maybe if there was sort of a, I don’t know, if someone you could contact, send an email, or ring or whatever so if you got any further questions or they want some more information that isn’t there”* (CS14).

Other frequent suggestions included providing other forms of program support, such as guidance videos introducing each module, the use of pre-completed examples, and tips on how to apply the skills learnt in participants’ daily lives. Two cancer survivor groups suggested a frequently asked question page, which was not mentioned by healthcare professionals or cancer support representatives.

#### Skill building

One element of the program praised by stakeholders was the inclusion of activities that build skills to help the user make lifestyle changes, rather than only providing information about what changes are required. All groups identified that this is especially helpful for developing mental health strategies (e.g., the mindfulness meditations and the thought record).

*“You’ve got the resources there and those mindfulness meditations if they are no longer than about, you know, three to four minutes then that's ideal. Especially for people that start doing it”* (CS07).

### Theme 3: relatability and relevance

Stakeholders emphasised that HLaC *Online* should normalise the after-treatment experience by including cancer-specific information and representative images of the diverse cancer survivor population.

#### Cancer-specific information

One concern frequently emphasised by all groups was ensuring that the program would be relatable and relevant to cancer survivors. It was important that the information and examples used within the program are cancer-specific.

*“So, perhaps this section might just need to be a bit more impactful for people with cancer. Perhaps a little bit less. I mean there’s some good things in there but maybe a bit more to kind of really connect it to a person with cancer what their experiences are”* (NGO02).

The need to normalise the survivorship experience was frequently identified by all stakeholder groups. Cancer survivors often discussed their own experience completing treatment and the emotional impact of no longer seeing oncology healthcare professionals as frequently, as well as the expectations from friends and family to quickly return to normal. All stakeholder groups felt strongly that this ‘new normal’ needed to be captured within the program.

Moreover, healthcare professionals more frequently identified the need for the program to include more education about the mental and physical impact of cancer and it’s associated treatment.

*“…I think it probably should be picked up somewhere in the program to acknowledge the side effects, the impact of the side effects and how to try to rectify them, or how to, yeah, work through them”* (HCP08).

Finally, all stakeholders endorsed including information about the benefits of engaging in a healthy lifestyle, particularly around reducing the risk of cancer- and treatment-related side effects.

*“And just, I guess educating them on what good choices are, what benefits do you get from eating this sort of food, rather than don't have this because it’s bad for you. Everyone knows that. It’s everywhere. You don’t need that… They are going to be thinking what can I be eating that’s gonna stop me from getting cancer again”* (HCP02).

#### Represent a diverse population

The stakeholders advocated that HLaC *Online* should include images that represent the diverse cancer survivor population, including representing the variety in age, gender, ethnicity, and levels of ability and fitness.

*“Yeah, so making maybe one of the start points or one of the picture representations a little bit more relatable to some of the people who aren’t very fit”* (HCP04).

#### Peer stories

Stakeholders reported it would be beneficial to include peer stories within the program. Short videos of peer stories were included in the wireframe in finding the new normal and the peer support modules. However, stakeholders suggested adding a peer support video into each of the main sections, so that users can relate to someone who has been through a similar experience and how they made changes to achieve a healthy lifestyle.

*“The videos with actual people telling their experiences, I think that is probably have the maximum impact. And because people will listen rather than kind of wade through loads and loads of text”* (HCP06).

*“People have, you know, someone to relate to. They sort of be like oh wow I went through that as well”* (CS14).

### Theme 4: navigating professional support

Navigating professional support covered the feedback relating to information about professional support access and providing links to additional resources.

#### Accessing professional support

All stakeholders emphasised the need for further information about professional support that is available to cancer survivors. Specifically, they suggested that information about how to access relevant health professionals and services was an important inclusion for each of the modules. This was particularly relevant to cancer survivors, who discussed their own experiences finding a mental health professional.

*“I mean I’ve found talking to my GP, he had trouble finding somebody that kind of. I mean I specifically wanted to try and talk someone that, you know, dealt with people that had cancer and could relate to a lot of the things. So, for me, I mean, it would be great if there was something very specific in there, you know, give me a guess a list of practitioners that dealt with that”* (CS04).

Further, the cancer survivor group were interested in providing more information about other supportive services and organisations, particularly in the areas of mental health.

*“And you have some links too for people [to] expand on if they need to. You know beyond blue or, you know, Black dog institute or whatever. So, having those numbers there and Lifeline all that. You know, having that there as backup underneath all of all of this stuff for people that are having dark thoughts”* (CS07).

#### Additional resources

The stakeholder groups suggested embedding links to credible information. Cancer survivors in particular emphasised that this program should be viewed as a starting point for healthy lifestyle change, and it should provide links to additional resources or mobile applications for users who wish to continue exploring ideas introduced in the program.

*“Look at what the Cancer Councils already got and put some links in to those resources would be really good idea to be supportive rather than reinvent the wheel”* (HCP08).

### Theme 5: peer and family support

The peer and family support theme encompassed (a) the stakeholders’ need to involve families in the program, both as a supporter of the cancer survivor and as individuals in need of support themselves and (b) to incorporate other various forms of peer support into the program.

#### Family support

Offering support for families within the program was strongly identified as a need by the healthcare professionals and cancer support representatives. They recommended providing support either via the cancer survivors’ user portal or by offering family members the opportunity to also sign up to use the program.

*“What about the carers and what about the family members? They would really benefit from this. If you can click it can go, I’m the parent I’m the patient slash I'm the carer. Because, if the carer can do this and understand their emotions, often a patient and carer or patient and loved one that are looking at one another for support”* (HCP03).

#### Peer support

Providing multiple avenues for peer support in the program was frequently identified by cancer survivors.

*“Because we all have different ways of looking for peer support. Some are one-on-one, some people like face-to-face support groups or can do it online, or sort of being online anonymously, you know, not like you and I, but where they can just use the discussion board. So, there’s a real wide variety of how people connect with a peer support group”* (CS11).

Cancer survivors provided recommendations for users to access peer support, often based on their own experiences of the peer support that they found helpful. These recommendations included face-to-face support (e.g., support groups) and Facebook groups. Healthcare professionals and cancer support representatives more frequently recommended peer support services offered by their organisations, such as Cancer Connect (a free telephone peer support service offered by various Cancer Councils).

## Discussion

This study fulfilled the *ideate* and *prototype* stages of the Design Thinking and Research Process co-design framework [30] by providing stakeholders with the opportunity to critique a prototype wireframe of the proposed HLaC *Online* program. Consistent with the first round of co-design, stakeholders continued to emphasise the importance of addressing mental health, fatigue management, and peer support [31]. However, the present study extended these previous findings and identified several new themes relating to program usability and support features: (a) specific website design considerations, (b) strategies for promoting and maintaining long-term user engagement, (c) enhancing relatability and relevance, (d) incorporating professional support, and (e) addressing the need for family and peer support.

A frequent observation made by all stakeholder groups was that maintaining engagement may pose a significant challenge to HLaC *Online*, a self-managed intervention. The majority of stakeholders described typical online program users as highly engaged within the first few weeks of a program, before gradually tapering off in interest and engagement. Consequently, the majority of the feedback focused on program features to encourage uptake and longer-term adherence to HLaC *Online*. These findings support previous investigations into engagement design features, which have consistently found that interventions should be easy to use, relevant to the target population, and include personalisation features, avenues for social support, and some level of guidance through, for example, reminders or a web-support contact [35, 36].

The stakeholder co-design process generated modifications to several aspects of the program, including simplifying activities viewed as too complex for a self-guided format, allowing consumers to self-select program reminder frequency, and providing further information on locating support from peers and healthcare professionals. These findings were induced and strengthened by the iterative nature of the co-design methodology, in which the current prototype was derived from the initial consultation of stakeholders, and prototype-feedback was then sought from that same group. As a result, stakeholders were enabled to provide guidance as to whether the needs identified in the first round of engagement had been sufficiently met and which needs required further consideration or development.

The involvement of different stakeholder groups, rather than a single group, enhanced the *ideate* and *prototype* stages of co-design [30]. Involving stakeholders who may be involved in the implementation of HLaC *Online* (e.g., through recommendation or program support) in addition to end-users enabled diverse feedback to be collated from cancer survivors, healthcare professionals, and cancer support representatives. Feedback provided by cancer survivor stakeholders largely focused on how to make the intervention relevant and accessible to the diverse cancer survivor population who will ultimately be the end-users of the program (i.e., through additional peer stories, different language settings, and printable options). In contrast, the healthcare professional and cancer support representatives drew from their expertise on how to best support users to make and sustain healthy lifestyle and long-term behaviour changes (i.e., beyond the intervention period of three months). This diversification of feedback ensured that suggested behaviour changes are accessible to all cancer survivors (e.g., focusing on walking instead of weighted exercises) and that it included information about the potential cancer- and treatment-related side effects that can complicate the behaviour change process. The benefit of including multiple stakeholder groups, particularly healthcare professionals and representatives from support organisations, has been noted in previous digital health intervention research [37].

Restrictions on stakeholders’ consultation time and limited cultural and professional diversity in the stakeholder group are two limitations of this study. Focus groups and interviews were time consuming, and engagement often felt rushed, especially with busy healthcare professionals. Consequently, stakeholders may have lacked adequate time to review each wireframe page in depth and only able to provide feedback based on their first impressions. Alternative co-design methodologies to reduce such time-constraints that could be considered in the future include providing the summary of the findings from the previous engagement and the wireframe ahead of engagement to allow more discussion time [33], or asking participants to complete and provide feedback on a set number of activities included in the program [32]. Further, the participant sample had inadequate representation of different cultures, such as Aboriginal and Torres Strait Islander Australians or Culturally and Linguistically Diverse (CaLD) Australians. Further developments made to HLaC *Online* based on current stakeholder feedback may not suit the needs of Aboriginal and Torres Strait Islander or other culturally diverse Australian cancer survivors. Future iterations of the HLaC *Online* program should consider engaging stakeholders from Aboriginal and Torres Strait Islander and other cultural group communities, to ensure the program is culturally safe and meets the unique needs of these communities. Additionally, the study may have been improved with involvement of website design experts (e.g., computer programmer and graphic designer), who may have provided additional ideas about what would work within the program which end-user stakeholder could provide their perspectives on. This limitation will be addressed in the next stage of program development, whereby website design experts will be involved in the development of the HLaC *Online* website*.*

Stakeholder feedback was integrated into the website design of HLaC *Online*. Key changes to the intervention design included offering HLaC *Online* with a responsive design for use on different devices, guidance videos to assist users completing activities, use of a mood rating as an alternative to the thought record, more information regarding the unique impacts of cancer and its treatment, guidance in each module on how to access relevant healthcare professions, and multiple options for accessing peer support. The feasibility and usability of this design iteration will be evaluated in a pre-post trial prior to testing the efficacy of HLaC *Online* via a randomised controlled trial.

In summary, continuing the co-design process through a second round of stakeholder engagement has further refined the development of HLaC *Online*. Specific feedback and advice provided by the stakeholder group has been incorporated to ensure that the content best meets the needs of cancer survivors and supports their undertaking of the self-guided intervention. Future development of digital health interventions utilising the co-design approach should explore alternative co-design methodologies that address the potential time constraints of the stakeholder group and consider the recruitment of multiple, culturally diverse stakeholder groups to ensure the proposed intervention best meets the needs and expectations of their target population.

## Supplementary Information

Below is the link to the electronic supplementary material.

Supplementary file1 (PDF 212 KB)

Supplementary file2 (PDF 156 KB)

Supplementary file3 (PDF 106 KB)

Supplementary file4 (PDF 133 KB)

## Data Availability

The datasets generated during and/or analysed during the current study are available from the corresponding author on reasonable request.
