# Supplementary material for: Co-designing Healthy Living after Cancer Online: an online nutrition, physical activity, and psychosocial intervention for post-treatment cancer survivors
Source: J Cancer Surviv. 2022 Nov 14;18(2):606–16. doi: 10.1007/s11764-022-01284-y (PMC9660094; doi:10.1007/s11764-022-01284-y)

Appendix 1 – Example pages from Healthy Living after Cancer Online wireframe

Figure 2.

Example home page from the HLaC Online wireframe

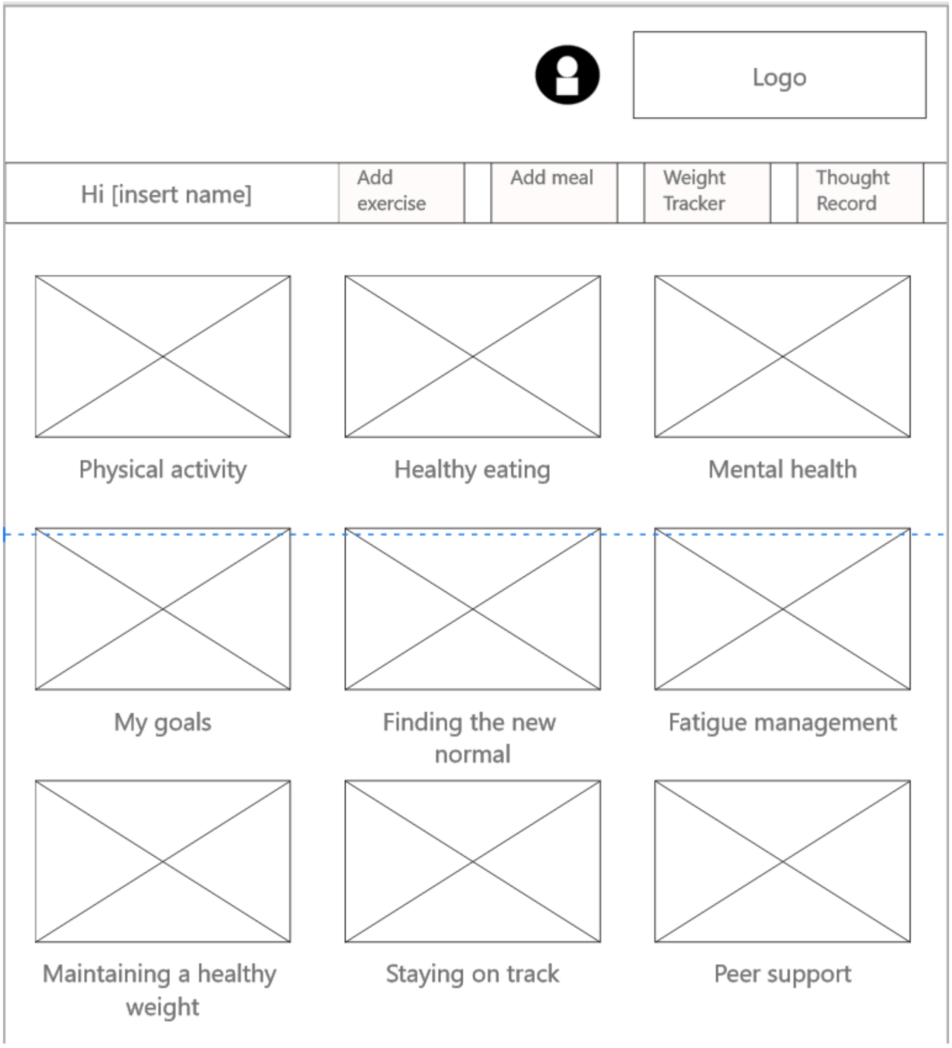

Figure 3.

Example thought record from HLaC Online wireframe

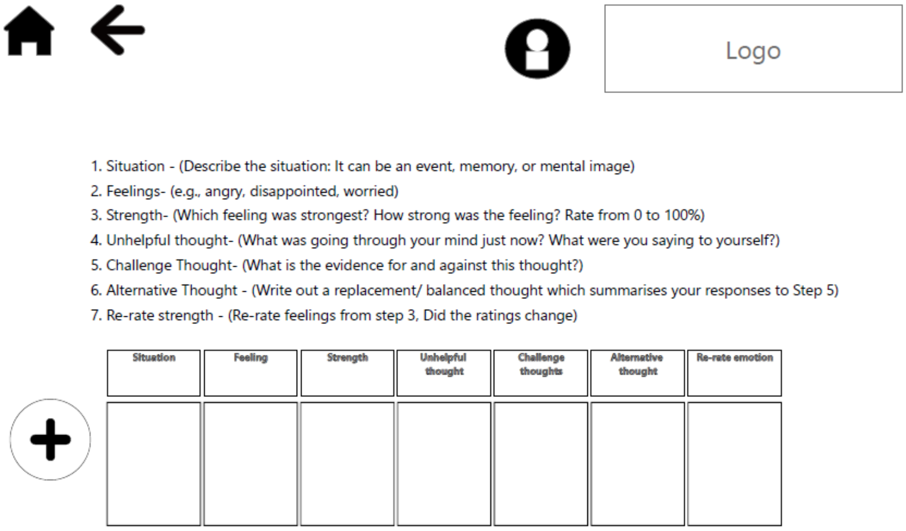

Figure 4

Example page from the physical activity module in Healthy Living after Cancer Online

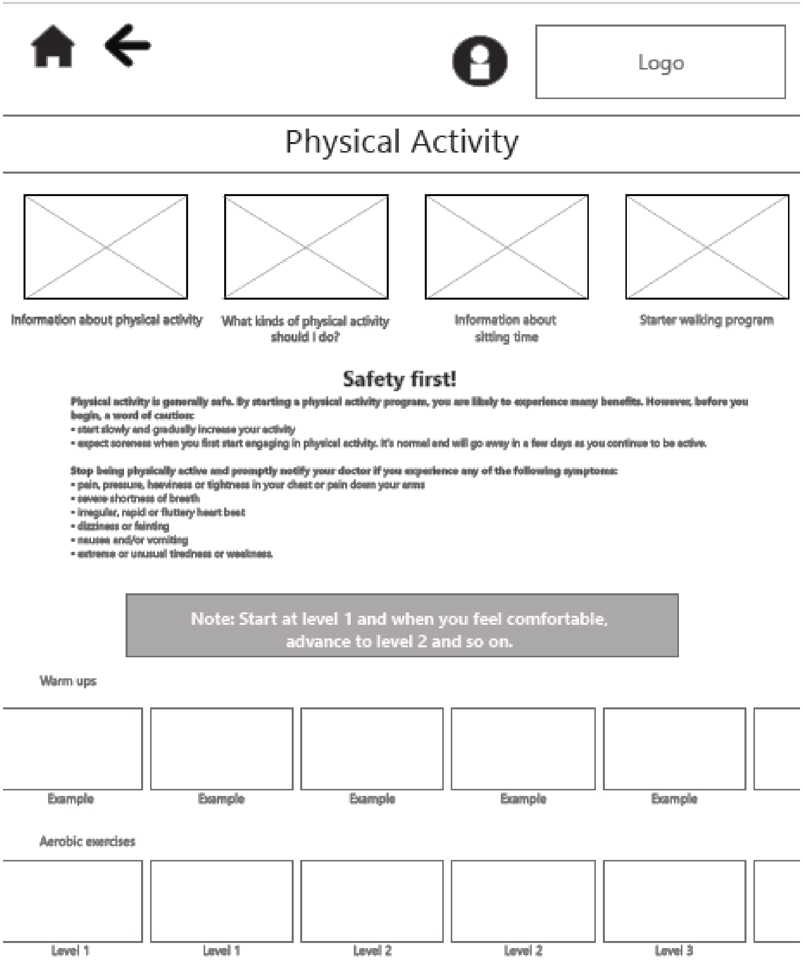

Supplement: Supplementary file 1 — Supplementary file1 (PDF 212 KB) [file 11764_2022_1284_MOESM1_ESM.pdf]
