# Supplementary material for: Co-designing Healthy Living after Cancer Online: an online nutrition, physical activity, and psychosocial intervention for post-treatment cancer survivors
Source: J Cancer Surviv. 2022 Nov 14;18(2):606–16. doi: 10.1007/s11764-022-01284-y (PMC9660094; doi:10.1007/s11764-022-01284-y)
Supplement: Supplementary file 2 — Supplementary file2 (PDF 156 KB) [file 11764_2022_1284_MOESM2_ESM.pdf]

Appendix 2 – topic guide for focus groups and interviews

Table 1.

Topic guide for the second round of stakeholder engagement focus groups and interviews

**Section 1: Findings from last focus group**
Powerpoint presentation about the key messages from round 1 of stakeholder engagement including the following:<br>1. Healthy living is defined as having good overall quality of life and includes physical health, mental health and adjustment to the new normal.<br>2. Healthy living programs should include mental health and peer support components and offer a flexible format with long term accessibility.
**Section 2: Wireframe of the online program**
Presentation of basic black and white template of the proposed program. Stakeholders were asked to provide their feedback on the home screen and modules with the following questions:<br>Home screen<br>• If you were signing into this page for the first time, how would you want it to look?<br>• What do you think of the layout?<br>• Do you think the important content readily available?<br>Modules<br>• What do you think of this page?<br>• Is this what you expected this section to look like?<br>• Is there anything else you would change or add?
**Section 3: How can users best be supported?**
Task to create a persona to represent a potential user of the program<br>• How could this person use this program to achieve their healthy living goals<br>• How often would they intend to use a program like this?<br>• What device would they use the program on?<br>• How could we support them in using this program?
