# Supplementary material for: Co-designing Healthy Living after Cancer Online: an online nutrition, physical activity, and psychosocial intervention for post-treatment cancer survivors
Source: J Cancer Surviv. 2022 Nov 14;18(2):606–16. doi: 10.1007/s11764-022-01284-y (PMC9660094; doi:10.1007/s11764-022-01284-y)
Supplement: Supplementary file 3 — Supplementary file3 (PDF 106 KB) [file 11764_2022_1284_MOESM3_ESM.pdf]

Appendix 3 – Example persona

Below is a summary of a persona created from the persona task in a focus group with three cancer survivors.

Persona description

Norma is a 53-year-old, female bowel cancer survivor who has recently completed cancer treatment, which included surgery, radiotherapy, and chemotherapy. Norma's healthy living goals include returning to an active lifestyle, to be able to play with her young grandchildren, and returning to work.

Description of how persona would use Healthy Living after Cancer Online

Norma would access the program on a variety of electronic devices, including a laptop, a tablet, and a smart phone. Norma uses the program as reference material and visit sections which are relevant to her meeting her goals, including physical activity, healthy eating, and maintaining a healthy weight. Norma would initially spend time on the website looking the information provided on the websites and she may be more engaged if provided with additional links to follow if she would like more information on a particular topic. Providing information is particularly important in the mental health section (e.g., information regarding common mental health concerns in cancer survivors), as Norma may need assistance with her emotional well-being. While she is not working, she may also choose to record her activity every few days. In this program, Norma would benefit from examples and tips on how to integrate different healthy habits into her lifestyle and information about how to access relevant healthcare professionals (e.g., a psychologist).
