# Supplementary material for: Co-designing Healthy Living after Cancer Online: an online nutrition, physical activity, and psychosocial intervention for post-treatment cancer survivors
Source: J Cancer Surviv. 2022 Nov 14;18(2):606–16. doi: 10.1007/s11764-022-01284-y (PMC9660094; doi:10.1007/s11764-022-01284-y)
Supplement: Supplementary file 4 — Supplementary file4 (PDF 133 KB) [file 11764_2022_1284_MOESM4_ESM.pdf]

Appendix 4 – Participant’s characteristics table

Tables 2

Participant characteristics of stakeholder group

	**Cancer survivors**		**HCP and NGO Representatives**	
	n	%	n	%
**Gender**				
Female	8	57.1	12	85.7
Male	6	42.9	2	14.3
**Most recent cancer diagnosis**				
Breast	6	42.9	-	-
Prostate	3	21.4	-	-
Rectal	2	14.3	-	-
Cervical	1	7.1	-	-
Hodgkin’s Lymphoma	1	7.1	-	-
**Profession or NGO representing**				
Nurse	-	-	7	53.8
Medical Oncologist	-	-	2	15.4
Representative from a non-governmental cancer support organisation	-	-	2	15.4
Clinical Psychologist			1	7.7
Physiotherapist	-	-	1	7.7
